# The impact of PET imaging on triple negative breast cancer: an updated evidence-based perspective

**DOI:** 10.1007/s00259-024-06866-9

**Published:** 2024-08-07

**Authors:** Luca Filippi, Luca Urso, Cristina Ferrari, Priscilla Guglielmo, Laura Evangelista

**Affiliations:** 1 Nuclear Medicine Unit, Department of Onco-hematology, Fondazione PTV Policlinico Tor Vergata University Hospital, Rome, Italy; 2 Department of Translational Medicine, University of Ferrara, Ferrara, Italy; 3 Nuclear Medicine Unit, Interdisciplinary Department of Medicine (DIM), University of Bari “Aldo Moro”, Bari, Italy; 4 Nuclear Medicine Unit, Humanitas Gavazzeni, Bergamo, Italy; 5 Nuclear Medicine Unit, IRCCS Humanitas Research Hospital, Rozzano, Italy; 6 Department of Biomedical Sciences, Humanitas University, Pieve Emanuele, Italy

**Keywords:** Triple negative breast cancer, PET/CT, Molecular imaging, Radiomics, Prostate specific membrane antigen, Theranostics

## Abstract

**Introduction:**

Triple-negative breast cancer (TNBC) is a subtype of breast cancer characterized by the absence of estrogen, progesterone, and HER2 receptors. It predominantly affects younger women and is associated with a poor prognosis. This systematic review aims to evaluate the current role of positron emission tomography (PET) in the management of TNBC patients and to identify future research directions.

**Methods:**

We systematically searched the PubMed, Scopus, and Web of Science databases up to February 2024. A team of five researchers conducted data extraction and analysis. The quality of the selected studies was assessed using a specific evaluation form.

**Results:**

Twenty-eight studies involving 2870 TNBC patients were included in the review. Key clinical applications of PET in TNBC included predicting pathological complete response (pCR) in patients undergoing neoadjuvant chemotherapy (NAC), assessing the prognostic value of baseline PET, and initial disease staging. Two studies utilized PSMA-ligand agents, while the majority used [^18^F]FDG-based PET. Significant associations were found between baseline [18F]FDG uptake and molecular biomarkers such as PDL-1, androgen receptor, and Ki67. Baseline [^18^F]FDG PET led to the upstaging of patients from stage IIB to stage IV, influencing treatment decisions and survival outcomes. In the NAC setting, serial PET scans measuring changes in [^18^F]FDG uptake, indicated by maximum standardized uptake value (SUVmax), predicted pCR with varying cut-off values correlated with different response rates. Semiquantitative parameters such as metabolic tumor volume (MTV) and PET lung index were prognostic for metastatic disease.

**Conclusions:**

In TNBC patients, [^18^F]FDG PET is essential for initial disease staging in both localized and metastatic settings. It is also useful for assessing treatment response to NAC. The ability of PET to correlate metabolic activity with molecular markers and predict treatment outcomes highlights its potential in TNBC management. Further prospective studies are needed to refine these clinical indications and establish its definitive role.

## Introduction

Breast cancer (BC) is a significant neoplasm with rapidly evolving management strategies over recent decades [1]. Triple-negative breast cancer (TNBC), characterized by the absence of detectable estrogen receptor (ER), progesterone receptor (PR), and human epidermal growth factor receptor 2 (HER2)/neu gene overexpression, accounts for 15–20% of all BC cases and predominantly affects younger patients, typically under 40 years old [2]. TNBC is associated with a higher recurrence rate and poorer prognosis compared to other BC subtypes, with a median survival of only 1–1.5 years following the diagnosis of metastatic disease [2, 3]. The lack of effective targeted therapies significantly limits the life expectancy of TNBC patients [4].

The landscape of TNBC treatment is rapidly evolving, driven by advances in molecular biology, immunotherapy, and precision medicine. Emerging targeted therapies, including PARP inhibitors, immune checkpoint inhibitors, and tyrosine kinase inhibitors, hold promise for improving outcomes in TNBC patients [5]. In example, clinical trials, such as OlympiAD and EMBRACA, have demonstrated the efficacy of PARP inhibitors such as Olaparib and Talazoparib in improving progression-free survival and objective response rates in advanced or metastatic TNBC patients with BRCA mutations [6]. In the KEYNOTE-522 trial, Pembrolizumab, demonstrated improved overall survival in TNBC patients with PD-L1-positive tumors [7]. However, despite significant progress in therapeutic and diagnostic approaches, several challenges persist in TNBC management. Indeed, the identification of novel targets, exploration of combination therapies, and development of predictive biomarkers are crucial for improving treatment outcomes.

Breast cancer frequently overexpresses glucose transporters GLUT 1–3, making it amenable to imaging with [^18^F]FDG PET/CT. However, different BC subtypes exhibit variable [^18^F]FDG avidity, reflecting differences in their glucose metabolism. Notably, TNBC and HER2-enriched BC typically show high [^18^F]FDG uptake, whereas luminal BC, particularly the luminal A subtype, exhibit faint [^18^F]FDG uptake [8]. However, a very recent published sub-analysis of the PHERGain trial highlighted the significant metabolic heterogeneity within HER2-enriched BC, depending on the expression of genes involved in cancer metabolism [9].

Currently, [^18^F]FDG PET/CT is commonly used in BC patients for initial staging or re-staging post-surgery or chemotherapy. Nonetheless, the most recent ESMO guidelines recommend [^18^F]FDG PET/CT only for specific clinical indications, such as inconclusive findings from conventional imaging [10]. Recently, international joint guidelines were released concerning the management of breast cancer patients undergoing [^18^F]FDG PET/CT across various disease settings. These guidelines highlight TNBC specifically, though they do not offer definitive recommendations for this subtype [11, 12].

The limited indications for [^18^F]FDG PET/CT in BC patients come from the heterogeneous literature, which often describes cohorts of mixed BC patients with varying subtypes and treatment regimens. Additionally, the high costs and limited availability of this imaging modality, given the high prevalence of BC, present further challenges. Despite these limitations, [^18^F]FDG PET/CT is valuable for the accurate systemic staging of selected patients with newly diagnosed BC. The detection of unsuspected extra-axillary regional nodal metastases and distant metastases can significantly impact staging, treatment planning, and prognosis [13].

Numerous studies utilizing PET/CT have attempted to categorize molecular subtypes of BC, assess the correlation between [^18^F]FDG uptake and various prognostic factors, and stratify the prognosis of TNBC [14–16]. These studies underscore the effective role of PET/CT in this patient category. Additionally, other radiopharmaceuticals, such as [^68^Ga]Ga-PSMA-11 and [^18^F]PSMA-1007, which target the prostate-specific membrane antigen (PSMA) overexpressed by TNBC-associated neovasculature, are emerging as potentially valuable tools [17]. These advancements also pave the way for approaches that combine diagnosis and therapy into a single platform, known as ‘theranostics.’

Moreover, radiomics, an emerging discipline that involves extracting quantitative and reproducible data from medical images (referred to as ‘features’), has shown promise in building predictive models for BC patients [18].

The aim of this systematic review is to summarize the current clinical evidence on the role of PET imaging in TNBC patients and to explore future directions in the application of molecular imaging in this oncological setting.

## Materials and methods

### Search strategy and study selection

A literature searches up to 29 February 2024 was conducted by interviewing the PubMed, Scopus, and Web of Science databases. The terms used were as follows: “triple negative breast cancer” or “TNBC” AND “PET/CT” or “positron emission tomography/magnetic resonance imaging.” The search was carried out with and without the addition of filters, such as English language only, type of article (original article, research article), and subjects (humans only). Preclinical studies and reports that included cohorts of patients with BC phenotypes other than TNBC (i.e., luminal A/B and/or HER2+) were not considered. Moreover, reviews, clinical reports, abstracts of meetings, and editorials were also excluded. Our goal was to provide an updated overview of the role of PET imaging in TNBC. Therefore, we selected only the papers that reflect the current state-of-the-art methodologies and used hybrid PET/CT or PET/MRI scanners. Additionally, for research produced by the same group, the reviewers carefully screened the papers; indeed, papers that reused the same patient cohort or primarily confirmed previously published results were considered ineligible. Three reviewers (L.U., P.G., C.F.) conducted the literature search, and two other reviewers (L.F., L.E.) independently selected the studies to consider, excluding duplicate papers. Any discrepancy was solved by a consensus. After combining all the records identified, the full texts were retrieved and further assessed by four of the reviewers (L.U., L.F., P.G., C.F.). One reviewer (L.E.) carefully checked the reference list of selected papers to detect any other potentially eligible studies. Considering the heterogeneity of the studies, a meta-analysis was not performed.

### Data extraction

General details were retrieved for each study considered, such as generic data (authors, journal name, year of publication and study design), patients’ characteristics (number of patients and their mean or median age), disease phase (i.e., staging or restaging), type of treatment, progression-free and overall survival. Selected imaging studies were analyzed using a modified version of the Critical Appraisal Skills Programme (CASP) (https://casp-uk.net/aboutus, accessed on 15 May 2024) checklist dedicated to systematic reviews. This system employs 10 questions focused on systematic reviews, aligning with the structure of our manuscript. Critical appraisal was performed by two reviewers (L.F. and L.E.), and discrepancies, if any, were solved by discussion with the other authors. Data extraction and quality assessment were done independently by three reviewers, and differences were solved by discussion.

## Results

**Fig. 1:**
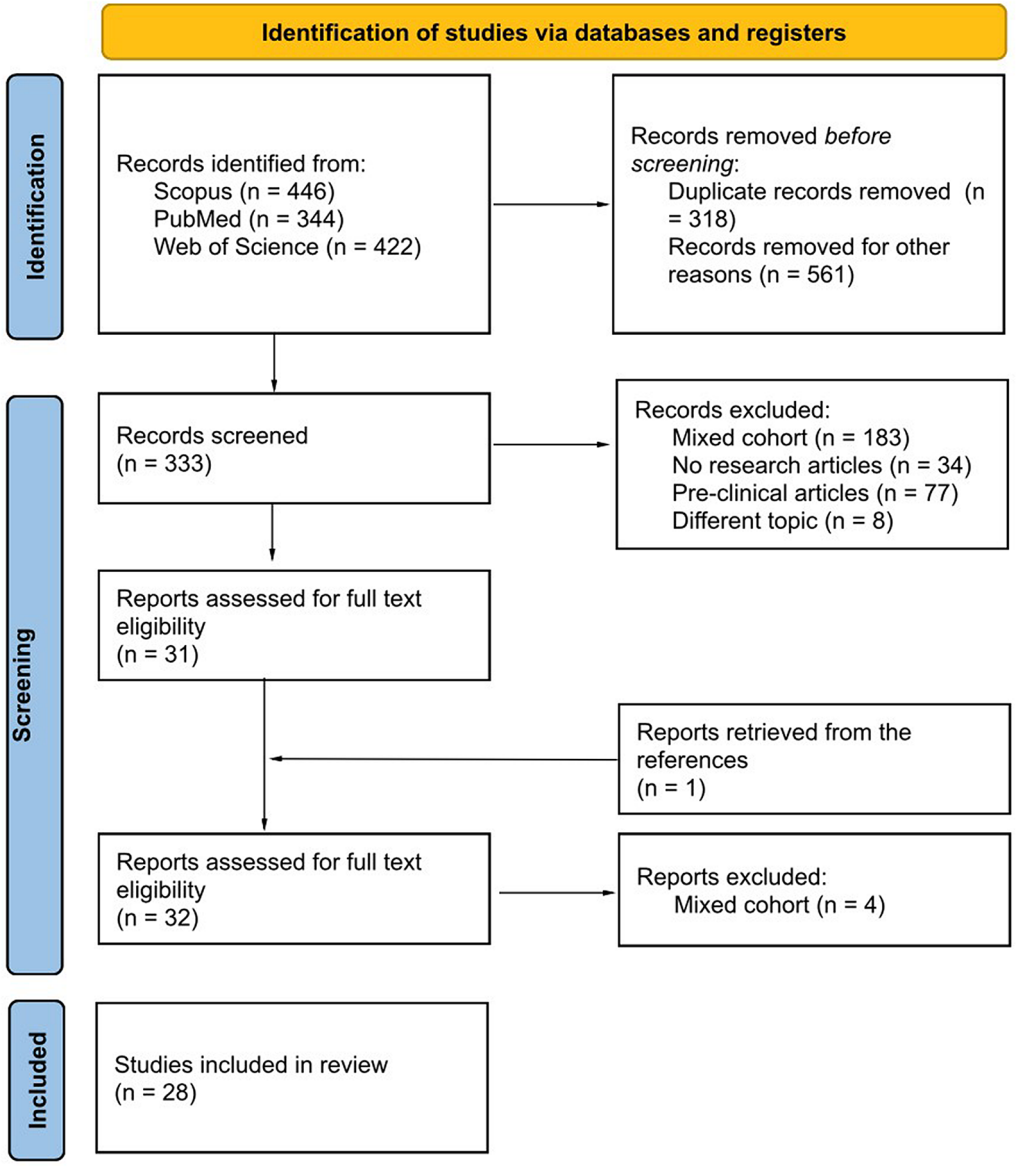
PRISMA flowchart indicating the selection process of the included studies

The resulting PRISMA search strategy is shown in Fig. 1. From the systematic literature search, twenty-eight papers were selected, including 2870 TNBC patients. Table 1 summarizes the main findings of the selected studies.

**Table 1:** Study and patients’ characteristics

Author	Year of publication	Main topic (or disease setting)	Study design	Number of patients	Radiopharmaceutical agent	Study analysis	Outcome	Topic
Koo et al. ^15^	2015	Baseline PET (correlation prognostic factors and [^18^F]FDG uptake)	R	103	[^18^F]FDG	DA	[^18^F]FDG uptake is correlated with Ki67 and tumor size	ki67 and [^18^F]FDG
Yue et al. ^26^	2015	Baseline PET (correlation between [^18^F]FDG uptake and prognosis)	R	200	[^18^F]FDG	SA	[^18^F]FDG can further stratify basal-like and non-basal like TNBC	Baseline staging
Marinelli et al. ^42^	2016	Metastatic PET (correlation between [^18^F]FDG uptake and prognosis)	R	47	[^18^F]FDG	SA	In metastatic TNBC, MTV seems a strong prognostic factor	Prognosis
Kim et al. ^43^	2017	Metastatic PET (correlation between [^18^F]FDG uptake and prognosis)	R	228	[^18^F]FDG	SA	Lymph node PET parameters are independent prognostic factors in TNBC	Prognosis
Tchou et al. ^19^	2009	Baseline PET (correlation prognostic factors and [^18^F]FDG uptake)	R	22	[^18^F]FDG	DA	[^18^F]FDG uptake correlates with Ki67	ki67 and [^18^F]FDG
Lee R et al. ^20^	2022	Baseline PET (correlation AR expression and [^18^F]FDG uptake)	R	608	[^18^F]FDG	DA	Low [^18^F]FDG uptake is a signature of AR expression	AR and [^18^F]FDG
Lee H et al. ^21^	2020	Baseline PET (correlation AR expression and [^18^F]FDG uptake)	R	156	[^18^F]FDG	DA	AR-positive TNBC show lower [^18^F]FDG than AR-negative	AR and [^18^F]FDG
Arslan E et al. ^58^	2023	Comparison between PSMA and [^18^F]FDG and correlation with Claudin expression in different clinical scenario	P	42	[^18^F]FDG and PSMA	DA	PSMA might be useful for theranostic ([^18^F]FDG higher uptake values than PSMA, but a precise diagnostic performance is not provided)	PSMA vs. [^18^F]FDG
Andryszak N et al. ^56^	2024	Head-to-head comparison between PSMA and [^18^F]FDG in different clinical scenario	P	10	[^18^F]FDG and PSMA	DP	Comparable uptake of both tracers in primary T and metastases, but PSMA superior in brain lesions	PSMA
Choi BB et al. ^27^	2018	Association between MRI features and [^18^F]FDG SUVmax	R	60	[^18^F]FDG	DA	Heterogeneous or rim enhancement, high signal on T2w and peritumoral edema were correlated with SUVmax	[^18^F]FDG vs. MR
Choi SH et al. ^22^	2018	Prognostic impact of PD-L1 expression and [^18^F]FDG uptake	R	145	[^18^F]FDG	SA	PD-L1 and tumor metabolism might have a role in predicting treatment failures	PDL-1 and [^18^F]FDG
Xie Y et al. ^25^	2022	Correlation between [^18^F]FDG uptake heterogeneity and immunotherapy outcome	R	32	[^18^F]FDG	SA	Baseline IETH predicts immunotherapy response	Heterogeneity and [^18^F]FDG
Groheux et al. ^30^	2012	Prognostic impact of change in [^18^F]FDG uptake after 2 cycles of NAC on pCR and DFS	P	20	[^18^F]FDG	SA	ΔSUVmax after 2 cycles is correlated with better prognosis	NAC and prognosis
Humbert et al. ^34^	2015	Prognostic impact of change in [^18^F]FDG uptake after 1 cycles of NAC on pCR	P	50	[^18^F]FDG	DA	ΔSUVmax after 1 cycle of NAC is a reliable predictor of pCR	NAC and response to therapy
Kiyoto et al. ^38^	2015	Prognostic impact of change in [^18^F]FDG uptake after NAC completion on pCR and DFS	R	32	[^18^F]FDG	SA	ΔSUVmax after NAC predicts pCR and DFS	NAC and prognosis
Groheux et al. ^35^	2016	Prognostic impact of change in [^18^F]FDG uptake after 2 cycles of NAC (2 different regimens) on pCR and EFS	P	78	[^18^F]FDG	SA	ΔSUVmax after NAC predicts pCR and EFS, however a different optimal cut-off was found for each distinct CHT regimen	NAC and prognosis
Groheux et al. ^40^	2018	Prognostic impact of baseline PET parameters and genomic features, as well as change in SUVmax after 2 cycles of NAC on pCR and EFS	R	55	[^18^F]FDG	SA	Baseline SUVmax combined with genomic grade index (GGI), as well as ΔSUVmax after NAC, predict pCR. ΔSUVmax after NAC and pCR are predictor of EFS.	NAC and prognosis
Basnet et al. ^37^	2020	Prognostic impact of change in SUVmax after 3 cycles of NAC on pCR	R	30	[^18^F]FDG	DA	A 50% in SUVmax after 3 cycles of NAC can be applied to discriminate responders vs. non–responders	NAC and response to therapy
Seban et al. ^32^	2023	Prediction of pCR after NAC or NACI according to baseline [^18^F]FDG PET parameters	R	191	[^18^F]FDG	SA	tumor SUVmax and total MTV were predictors of pCR at both univariate and multivariate analysis in both NAC and NACI cohorts	NAC and response to therapy
Bouron et al. ^33^	2021	Prediction of pCR after NAC according to baseline [^18^F]FDG PET conventional and textural parameters	R	74	[^18^F]FDG	SA	Conventional and textural parameters from baseline [^18^F]FDG PET failed to predict pCR after NAC	NAC and response to therapy
Humbert et al. ^41^	2016	Prognostic relevance of tumor blood flow changes (DBF) at dynamic [^18^F]FDG PET in TNBC patients treated with NAC	P	46	[^18^F]FDG	SA	A decrease in DBF (between baseline and post 1 cycle of NAC), as a surrogate of tumor perfusion and neoangiogenesis, is an incremental prognostic stratification when combined with pCR.	NAC and prognosis
Groheux et al. ^39^	2015	Prognostic stratification of TNBC patients according to the combined results of baseline [^18^F]FDG PET and pathology after NAC	P	85	[^18^F]FDG	SA	The results of baseline [^18^F]FDG PET combined with those of pathology after NAC allow to stratify TNBC patients into 3 subgroups with different prognosis	NAC and prognosis
Ulaner et al. ^28^	2016	Baseline PET/CT for early stage TNBC (< III)	R	232	[^18^F]FDG	DA/SA	PET/CT upstaged 15% of patients with initial stage IIB TNBC, demonstrating unsuspected distant metastases, with impact on management and survival	Baseline staging
Kimura et al. ^23^	2023	Baseline SUVmax could provide information regarding tumor immune microenvironment (TIME) components in TNBC patients, as favorable prognostic factors and predictive biomarkers for neoadjuvant CHT	R	54	[^18^F]FDG	DA	Baseline SUVmax was significantly correlated with CD8/FOXP3 ratio, which results the only independent predictive factor for pCR at multivariate analysis --> [^18^F]FDG PET/CT might help predict the effects of NAC in TNBC:	NAC and response to therapy
Xie et al. ^45^	2019	Lung Metastatic PET (prognostic value of tumor heterogeneity and first-line platinum-based therapy response)	R	31	[^18^F]FDG	SA	Intratumor heterogeneity assessed by lung index (LI) and TLG of mTNBC on baseline PET/CT scans have predictive value for treatment outcome and OS.	Metastastic staging
Gong et al. ^46^	2018	Metastatic PET (prognostic value of tumor heterogeneity and first-line platinum-based therapy response)	R	42	[^18^F]FDG	SA	Heterogeneity index (HI) and MAX among metastatic lesions, especially in visceral lesions, on baseline PET/CT have predictive value for treatment response.	Mtastastic staging
Romeo et al. ^48^	2022	Discrimination of TNBC from other BC subtypes using a ML model trained with [^18^F]FDG PET/MRI radiomic data	P	86	[^18^F]FDG	DP	Some of the models proposed showed high accuracy in discriminating TNBC from other BC subtypes	Radiomic
Bouron et al. ^49^	2022	Association between metabolic, volumetric and textural parameters measured at baseline [^18^F]FDG PET/CT and survival outcomes (disease-free survival and overall survival) in patients with non-metastatic TBNC	R	111	[^18^F]FDG	SA	Textural features associated with metabolic and volumetric parameters of baseline [^18^F]FDG PET/CT have a prognostic value for identifying high-relapse-risk groups in early TNBC patients	Radiomic

R: retrospective; P: prospective; DP: diagnostic performance; DA: descriptive analysis; SA: statistical analysis

Based on the CASP analysis, the quality of the study was variable (Table 2). The statement relative to the aims of the research was clear in all the selected studies. However, the quality of the methodology was appropriate in 8/27 (30%) papers. Indeed, the study design was unclear in many cases (*n* = 9, 33%). The data was collected in accordance with the research issue in all studies. The ethical statement was released in 18 (67%) reports and the value of each study was low in 3 (11%), moderate in 6 (22%) and high in 16 (59%). In 2 cases the value was considered potential.

**Table 2:** CASP assessment of the included studies

Author	Was there a clear statement of the aims of the research?	Is a qualitative methodology appropriate?	Was the research design appropriate to address the aims of the research?	Was the recruitment strategy appropriate to the aims of the research?	Was the data collected in a way that addressed the research issue?	Has the relationship between researcher and participant been adequately considered?	Have ethical issues been taken into consideration?	Was the data analysis sufficiently rigorous?	Is there a clear statement of findings?	How valuable is the research?
Koo et al. ^15^	Yes	Yes	Can’t tell	Can’t tell	Yes	Can’t tell	Yes	Yes	Yes	Moderate
Yue et al. ^26^	Yes	Yes	Can’t tell	Can’t tell	Yes	Can’t tell	Yes	Yes	Yes	Moderate
Marinelli et al. ^42^	Yes	Yes	Can’t tell	Can’t tell	Yes	Can’t tell	Yes	Yes	Yes	Highly valuable
Kim et al. ^43^	Yes	Yes	Yes	Yes	Yes	Can’t tell	Yes	Yes	Yes	Highly valuable
Tchou et al. ^19^	Yes	No	Can’t tell	Can’t tell	Yes	Can’t tell	Yes	Yes	Yes	Moderate
Lee R et al. ^20^	Yes	Yes	Yes	Yes	Yes	Can’t tell	Yes	Yes	Yes	Highly valuable
Lee H et al. ^21^	Yes	No	Can’t tell	Can’t tell	Yes	Can’t tell	Yes	Yes	Yes	Moderate
Arslan E et al. ^58^	Yes	No	Can’t tell	Can’t tell	Yes	Can’t tell	Yes	Yes	Yes	Low (small number of patients)
Andryszak N et al. ^56^	Yes	No	Can’t tell	Can’t tell	Yes	Can’t tell	Yes	Yes	Yes	Low (small number of patients)
Choi BB et al. ^27^	Yes	Yes	Can’t tell	Can’t tell	Yes	Can’t tell	Yes	Yes	Yes	Moderate
Choi SH et al. ^22^	Yes	Yes	Can’t tell	Can’t tell	Yes	Can’t tell	Yes	Yes	Yes	Highly valuable
Xie Y et al. ^25^	Yes	No	Yes	Yes	Yes	Can’t tell	No	Yes	Yes	Highly valuable
Groheux et al. ^30^	Yes	No	Yes	Yes	Yes	Can’t tell	No	Yes	Yes	Highly valuable
Humbert et al. ^34^	Yes	No	Yes	Yes	Yes	Can’t tell	No	Yes	Yes	Highly valuable
Kiyoto et al. ^38^	Yes	No	Yes	Yes	Yes	Can’t tell	No	Yes	Yes	Highly valuable
Groheux et al. ^35^	Yes	No	Yes	Yes	Yes	Can’ t tell	No	Yes	Yes	Highly valuable
Groheux et al. ^40^	Yes	No	Yes	Yes	Yes	Can’ t tell	Yes	Yes	Yes	Highly valuable
Basnet et al. ^37^	No	No	Yes	Yes	Yes	Can’ t tell	No	No	Can’t tell	Moderate
Seban et al. ^32^	Yes	No	Yes	Yes	Yes	Can’ t tell	Yes	Yes	Yes	Highly valuable
Bouron et al. ^33^	Yes	No	Yes	Yes	Yes	Can’ t tell	Yes	Yes	Yes	Moderate, in contrast with other reports
Humbert et al. ^41^	Yes	No	Can’t tell	Yes	Yes	Can’ t tell	No	Can’t tell	Yes	Low due to the small cohort
Groheux et al. ^39^	Yes	No	Yes	Yes	Yes	Can’ t tell	Yes	Yes	Yes	Highly valuable
Ulaner et al. ^28^	Yes	Yes	Yes	Yes	Yes	Can’ t tell	Yes	Yes	Yes	Highly valuable
Kimura et al. ^23^	Yes	No	Yes	Yes	Yes	Can’ t tell	No	Yes	Yes	Highly valuable
Xie et al. ^45^	Yes	No	Yes	Yes	Yes	Can’ t tell	Yes	Yes	Yes	Highly valuable
Gong et al. ^46^	Yes	No	Yes	Yes	Yes	Can’ t tell	Yes	Yes	Yes	Highly valuable
Romeo et al. ^48^	Yes	No	Yes	Yes	Yes	Can’ t tell	No	Yes	Yes	Potentially valuable
Bouron et al. ^49^	Yes	No	Yes	Yes	Yes	Can’ t tell	Can’t tell	Yes	Yes	Potentially valuable

### Qualitative analysis

Among the 28 selected studies, twenty (71.4%) were retrospective, with only a minority (*n* = 8, 28.6%) being prospective. Evaluation of the available data revealed heterogeneity across the various examined clinical settings (e.g., baseline staging, assessment of treatment response and assessment of recurrence) and to a lesser degree in the tracers employed for PET imaging. Notably, only two studies utilized PSMA-ligand, while the remaining utilized [^18^F]FDG.

The selected articles were subdivided according to the clinical settings described in the joint EANM‑SNMMI guideline for [^18^F]FDG PET/CT imaging in BC [11]. The most extensively investigated area was the potential of [^18^F]FDG PET/CT for predicting pathological complete response (pCR) in TNBC patients undergoing NAC (*n* = 10). This was followed by studies on the prognostic value of baseline PET/CT (*n* = 5) and initial staging of disease (*n* = 4). Four studies explored the correlation between [^18^F]FDG uptake in tumors and the expression of TNBC-correlated biomarkers such as androgen receptor (AR) or programmed death protein ligand-1 (PDL-1). Additionally, one study compared the performance of [^18^F]FDG PET/CT with that of MRI. Finally, two papers investigated the potential of PET-based radiomic approaches in TNBC and two more used PSMA-ligand PET/CT and will be presented in separate sub-sections.

**Fig. 2:**
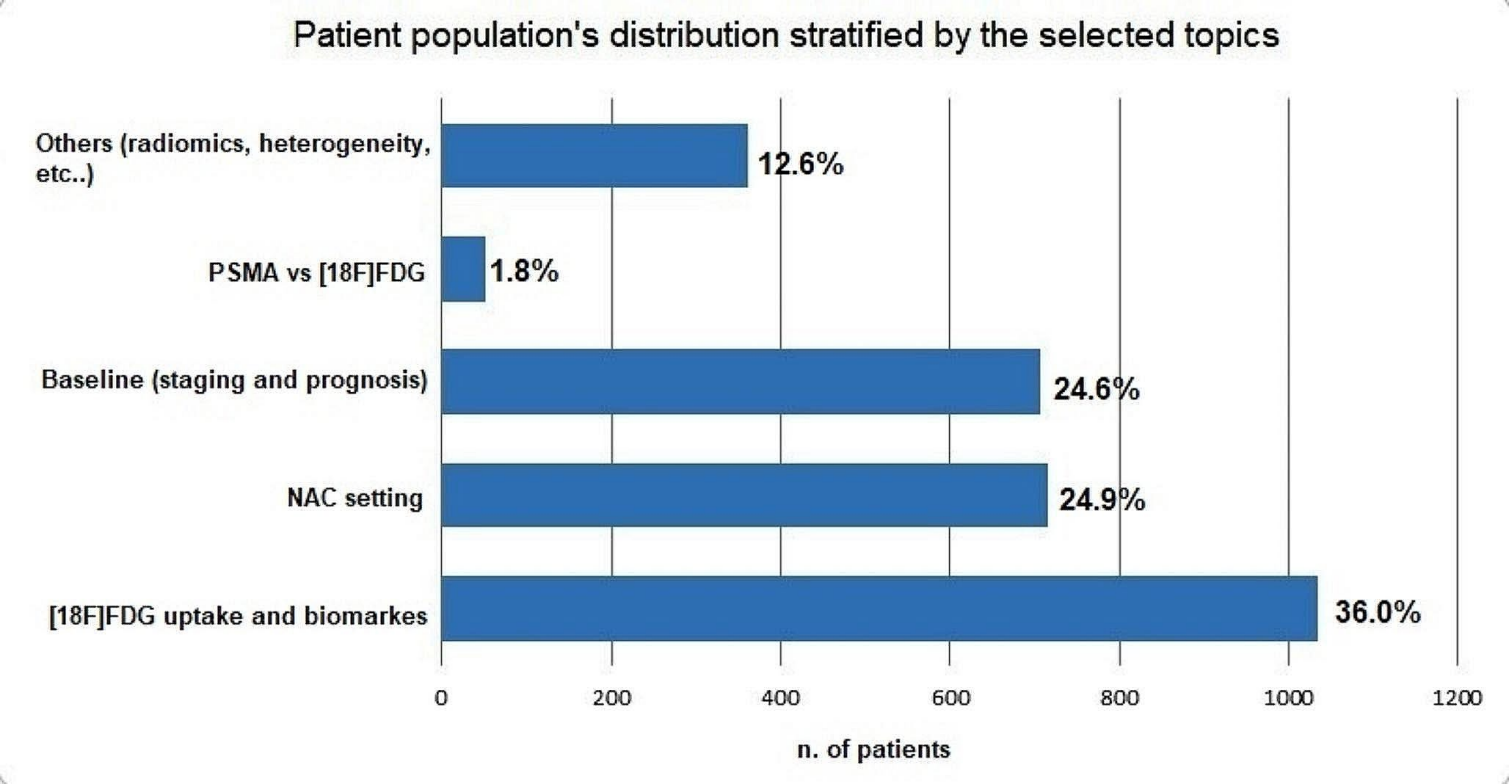
Graphical representation of the distribution of the entire population of selected studies in relation to the different thematic macro-areas

The entire patient population was distributed into the following macro areas: (1) correlation between [^18^F]FDG uptake and biomarkers’ expression (AR, Ki67, PDL-1), including 1034 patients (36.0%); (2) NAC setting, encompassing 715 patients (24.9%), covering response prediction, response monitoring, and prognostic stratification; (3) baseline and prognosis, consisting of 707 patients (24.6%), merging baseline staging and prognosis; (4) PSMA-PET, including 52 patients (1.8%); and (5) ‘Others,’ which includes [^18^F]FDG vs. MRI, heterogeneity and [^18^F]FDG, metastatic staging, and radiomic studies, accounting for 362 patients (12.6%) (Fig. 2).

In the selected works, various methodological approaches for reporting the results were found. Specifically, in 9 papers (32.25%), a summary statistic quantitatively describing the distribution of the observed features in the analyzed population (descriptive analysis/DA) was used. In 16 manuscripts (57.25%), statistical analysis (SA) approaches such as Kaplan-Meier analysis and univariate and multivariate analysis were used to define the prognostic impact of PET in different contexts. One manuscript (3.5%) employed a mixed DA + SA approach, and 2 papers (7%) focused on describing the diagnostic performance of PET imaging.

The key findings of the selected manuscripts for each of the aforementioned thematic areas are summarized in the following paragraphs.

### Baseline evaluation and staging

Information provided by a baseline [^18^F]FDG PET/CT scan is particularly significant in patients with TNBC. Indeed, [^18^F]FDG uptake can serve as both a predictive and a prognostic biomarker. The prediction of therapy response is a complex concept, but imaging can contribute valuable insights. Several studies have shown that baseline [^18^F]FDG uptake in primary TNBC can be associated with the expression of molecular targets such as PDL-1 and the androgen receptor (AR), or correlated with the proliferation index Ki67.

Koo et al. [15] and Tchou et al. [19] both described a significant correlation between [^18^F]FDG uptake and Ki67 in 103 and 22 patients, respectively. Additionally, studies by Lee R. [20] and Lee H. [21] demonstrated that lower [^18^F]FDG uptake predicted AR expression, suggesting potential for alternative treatments. Similarly, Choi et al. [22] found a correlation between high [^18^F]FDG uptake and PDL-1 expression, proposing the use of immunotherapy in TNBC, which is often resistant to chemotherapy.

Kimura and colleagues [23] investigated whether the metabolic parameter SUVmax could provide information regarding the tumor immune microenvironment (TIME) components in TNBC patients. They noted that SUVmax significantly correlated with the CD8/forkhead box P3 (FOXP3) ratio, an immune-functional marker of tumor-infiltrating lymphocytes (TILs), which are major components of TIME and are substantially related to tumor progression and response to chemotherapy in BC [24]. A high CD8/FOXP3 ratio corresponded to a high SUVmax.

Moreover, Xie et al. [25] reported a correlation between [^18^F]FDG uptake and tumor heterogeneity, which can be associated with varying responses to immunotherapy. However, Yue at [26] demonstrated that baseline [^18^F]FDG PET/CT has a highly ability to differentiate between basal and non-basal like histology, underlying the power of molecular imaging to predict the prognosis of this aggressive disease. Also, Choi [27] found a correlation between [^18^F]FDG uptake and MRI features, demonstrating that peritumoral edema was associated with high [^18^F]FDG uptake, indicating a more aggressive disease.

These findings underscore the multifaced role of [^18^F]FDG PET/CT in TNBC, providing valuable information for predicting treatment responses and understanding tumor biology.

Among the few studies with large and homogeneous cohorts, Ulaner et al. [28] retrospectively evaluated the role of baseline [^18^F]FDG PET/CT in 232 patients with newly diagnosed stage I-IIIC TNBC. The study demonstrated that a significant percentage (15%) of patients were upstaged by [^18^F]FDG PET/CT from initial stage IIB to stage IV. This upstaging had a notable impact on treatment management and was associated with significantly shorter survival, with a 3-year Kaplan-Meier estimate of 0.33 (95% CI 0.13–0.55) compared to 0.97 (95% CI 0.76–0.93) for those who were not upstaged (*p* < 0.0001) [28].

Additionally, the site of metastases varied by primary tumor subtype, with TNBC patients more frequently exhibiting extra-skeletal metastases than those with ER+/HER2- tumors [29, 30]. N3 disease was also more common in patients with grade 3 tumors and TNBC subtype [30]. Given the high proportion of extra-skeletal metastases in TNBC, staging with [^18^F]FDG PET/CT could be beneficial for these patients at least as early as stage IIB.

### Assessment and prediction of treatment response

The largest amount of data regarding the use of [^18^F]FDG PET/CT in this clinical setting are relative to the assessment of response after NAC. Among those, we can subdivide the studies according to their primary end-points in the following 3 groups: (1) evaluate [^18^F]FDG PET/CT to predict the response to NAC, (2) assess the utility of [^18^F]FDG in evaluating an early response to treatment, by using serial PET scans and (3) determine if [^18^F]FDG PET/CT can have a prognostic role in this setting.

Starting from the first end-point, it has been well established that pCR after NAC is a strong prognostic factor in BC patients, including those affected by TNBC [31]. However, it is still debated which [^18^F]FDG PET parameters can be useful to predict pCR. Seban et al. [32] demonstrated that a high SUVmax and a low MTV, referring to the primary tumor, were predictive of pCR in both univariate and multivariate analyses, across patients treated with either NAC or NAC plus immunotherapy (NACI). However, their results were in contradiction to those previously reported by Bouron et al. [33]. The two studies, conducted by Seban’s and Bouron’s groups [32, 33], reported significantly different rates of pCR. In the first study, pCR was achieved in 50% of patients who received NAC and in 73% of patients who received NACI. In contrast, the second study reported a pCR rate after NAC of only 29.7%.

For the second end-point, different experiences have been reported [34–38]. Indeed, in some cases, a baseline PET was made after including a second scan after a various number of cycles of neoadjuvant therapy. Humbert and colleagues [34] found that the mean ΔSUVmax, obtained from baseline to the first cycle of therapy, was 43.7 ± 25.4% for primary tumors and 50.1 ± 29.2% for metastatic lymph nodes. They also observed a significant difference in ΔSUVmax between patients who achieved pCR after NAC and those who did not. Furthermore, ΔSUVmax emerged as the only independent predictor of pCR in multivariate analysis. Groheux et al. [36]. demonstrated that the mean change in [^18^F]FDG uptake between baseline and interim PET (after 2 cycles of therapy), measured by SUVmax (ΔSUVmax), was found to be -45%. The same authors [35], after 4 years and by including more patients (*n* = 78 patients vs. 20 patients) confirmed that ΔSUVmax (Calculated between baseline PET and that after 2 cycles of NAC) was more pronounced in patients achieving pCR. Basnet and coworkers [37] investigated the role of [^18^F]FDG PET carried out at baseline and after 3 cycles of NAC in 30 TNBC patients. A reduction of more than 50% in SUVmax within the target lesion was used to discriminate responders from non-responders. Finally, in a study by Kiyoto et al. [38], PET/CT was used before starting and after the last cycle of NAC, demonstrating a relevant difference in ΔSUVmax between patients achieving pCR and those who did not, with 81.3% identified as the optimal ΔSUVmax threshold to differentiate between the two groups. In statistical analysis, N-stage, clinical stage, and ΔSUVmax emerged as significant predictors of pCR.

For the last end-point relative to the prognostic meaning of [^18^F]FDG PET/CT in patients undergoing NAC, some conclusions were drawn. Groheux and colleagues [39] identified three groups of patients: patients without distant metastases at baseline [^18^F]FDG PET/CT who achieved pCR after NAC; patients with metastases at distance at baseline [^18^F]FDG PET/CT who achieved pCR after NAC; patients with metastases at distance at baseline [^18^F]FDG who did not achieve pCR after NAC. Interestingly, disease specific survival (DSS) differed significantly among the three subgroups (log-rank *P* < 0.001), allowing to stratify patients’ prognosis. The same group [39] also found a correlation between the changes in [^18^F]FDG uptake before and after 2 cycles of therapy and prognosis. ROC analysis revealed that the optimal ΔSUVmax cut-off for predicting DFS was 42%. When patients were divided based on this ΔSUVmax cut-off, metabolic responders (reduced ΔSUVmax ≥ 42%) exhibited significantly longer DFS compared to non-metabolic responders (reduced ΔSUVmax < 42%). Again, the same research team [40], by analyzing different parameters, showed that only ΔSUVmax (calculated between baseline PET scan and that after 2 cycles of NAC) with a cutoff value of 50% (*P* = 0.048) and pathological response (*P* = 0.014) emerged as significant predictors of EFS. Kiyoto and colleagues [38] dichotomizing patients based on ΔSUVmax (pre- and post-therapy PET scan), reported that a significant discrepancy in DFS became apparent between those having a ΔSUVmax ≥ 81.3% vs. <81.3%. However, the prognostic value provided by ΔSUVmax is relative to both a specific cut-off and to the type of treatment, as demonstrated by Groheux et al. [35]. Finally, Humbert et al. [41], using a dynamic PET acquisition at baseline and after 2 cycles (interim PET) of therapy, showed that a persistent or increased tumor vascularization on the interim PET was independently associated with a short OS.

To date, no studies assessing response to systemic therapies with [^18^F]FDG PET/CT in metastatic TNBC has been published in literature.

Conversely, some few papers explored the role of [^18^F]FDG PET/CT in metastatic TNBC patients, focusing mainly on the prognostic relevance of semiquantitative parameters. In metastatic TNBC, semiquantitative data extracted from [^18^F]FDG PET/CT, such as MTV, TLG and others, resulted as prognostic factors [42–44]. In addition to traditional PET parameters, new measures of disease heterogeneity, especially among metastatic sites, are proposed in literature within this context [44]. With regards to lung metastases, Xie et al. [45]. suggested PET lung index (LI) as a new simple and noninvasive “biopsy” method for targeted metastasis that determines tumor heterogeneity with predictive value for response to therapy and OS. Baseline low LI (< 0.56) and TLG (< 3.54) values resulted predictors of better treatment outcome and PFS and could help clinicians in identifying patients with a good prognosis. Moreover, while LI resulted an independent predictor of PFS, low TLG resulted in an independent predictor of OS [45]. Furthermore, considering that metastatic lesions could have completely different tumor biology and metabolism compared with primary lesions or between each other, Gong et al. [46]. collected the highest (MAX) and lowest (MIN) value of [^18^F]FDG uptake and Heterogeneity index (HI) – measured by dividing MAX and MIN – across total metastatic lesions (-T), visceral metastatic lesions (-V) and non-visceral metastatic lesions (-N). Both high MAX-T (> 10.5) and high HI-T (> 1.9) were significant predictive factors for shorter PFS, whereas OS was only associated with MAX-T. Furthermore, MAX and HI have greater potential to predict PFS when measured in visceral lesions rather than non-visceral lesions, as visceral involvement had a greater impact on patient survival [46].

### Assessment of recurrence

According to our systematic research, no papers investigating the role of [^18^F]FDG PET/CT in TNBC patients with suspicious recurrence have been published yet.

### Radiomics

In recent years, radiomics has significantly assumed importance in the scientific literature. Texture analysis is useful to investigate tumor heterogeneity, which is particularly relevant in TNBC rather than in other BC subtypes, reflecting its malignant potential [47]. Baseline [^18^F]FDG PET/CT parameters and texture analysis could non-invasively characterize biological tumor heterogeneity and predict treatment outcome, with highly heterogeneous [^18^F]FDG uptake disease likely to be closely related to multidrug resistance [45, 46]. Some additional acknowledgments provided by radiomics have been released in TNBC, although still very preliminary and in a limited population (*n* = 197 patients). The only available papers from Romeo et al. [48] and Bouron et al. [49], described the ability of radiomics by [^18^F]FDG PET/CT images in discriminating between TNBC and other histopathologies, and in defining the prognostic role of additional features in this population, respectively. In particular, in the study from Bouron and coworkers [49], some textural features associated with metabolic and volumetric parameters were found to be important predictive factors of relapse in TNBC, potentially useful for identifying high-risk patients.

### PSMA vs. [^18^F]FDG

Prostate-specific membrane antigen (PSMA) is a type 2 transmembrane glycoprotein highly expressed in a vast majority of prostate cancer (PCa) cells [50]. Alongside PCa, elevated PSMA expression associated with neovascularization has also been reported in many non-prostatic malignant solid tumors, including BC [51–53]. In particular, [^68^Ga]Ga-PSMA PET/CT scans demonstrated a greater incidence of PSMA positivity in TNBC patients in contrast to Luminal A subtype [54, 55]. Moreover, to further investigate the added value of PSMA-based PET/CT in this clinical scenario, two papers have been published. In both cases, the comparison with [^18^F]FDG was reported, with the following conclusions: (1) [^18^F]PSMA-1007 can detect more lesions than [^18^F]FDG, mainly in the skeleton, whereas brain metastases can be exclusively detected by [^18^F]PSMA-1007 [56] and (2) [^68^Ga]Ga-PSMA-11 can be correlated with Clau 4 that is expressed in epithelia and endothelia of cancer cells that are associated with metastasis and poor prognosis in BC patients [57] and with a high ki67 index, thus opening the way for implementing a theranostic approach also in TNBC patients [58].

## Discussion

In TNBC, baseline [^18^F]FDG PET/CT should be considered both a predictive and prognostic biomarker. Indeed, as emerged from the available literature data, [^18^F]FDG uptake can be correlated with the expression of various markers that are linked with the aggressive behavior of these tumors and may also guide the selection of patients to treat with specific targeted therapies. AR expression, PDL-1 and tumor microenvironment can be useful for selecting patients who could be candidates to AR-inhibitors, immunotherapy or other drugs that can effectively act against the cancer.

In the initial staging of disease, [^18^F]FDG PET/CT can significantly change the stage of the tumors, mainly in patients with stage II/III. Similarly, to what has been described for PSMA PET in prostate cancer, the deriving “Will-Rogers phenomenon” can dramatically change patients’ prognosis, mainly when the disease is upstaged. However, until now only few data are available about the impact of PET/CT on survival outcomes [28]. Nevertheless, according to the ESMO guidelines [10], PET/CT is now suggested in patients with locally advanced TNBC, because of its impact on the therapeutic management. However, further findings should be collected in the future for solving the issue of survival rate by introducing the next generation imaging in the diagnostic flow-chart.

In addition to the visual assessment, semiquantitative analysis is always fascinating for the interpretation of [^18^F]FDG PET/CT imaging. For many years, SUVmax has been the most clinically used PET parameter for prognostication and response monitoring. However, despite its widespread use and validation, SUVmax has limitations. It is prone to measurement errors due to the partial volume effect and only reflects the hottest part of the lesion, lacking comprehensive tumor biology information. To address these limitations, volumetric parameters like MTV and TLG have been introduced [59]. Furthermore, the introduction of simple software for the calculation of some PET parameters, such as whole-body values, has significantly increased the interest for these variables. Data about TNBC are limited to patients with metastatic disease [60, 61]. As emerged from the available studies, the amount of glucose uptake in the whole metastatic lesions is associated with the prognosis, thus contributing to stratify patients with a poor outcome as compared to the counterpart.

Regarding the role of [^18^F]FDG PET in assessing the response of TNBC to NAC through serial PET/CT scans, several considerations can be made. Firstly, there was notable heterogeneity across the selected studies concerning the optimal timing for follow-up / re-staging PET/CT. Some studies [34, 36] suggested that evaluating the metabolic response is feasible as early as after 1–2 cycles of therapy, and changes in SUVmax (ΔSUVmax) can be correlated with pCR. Additionally, while there is no consensus on the exact cut-off to differentiate responders from non-responders, a ΔSUVmax of -45/50% may be adequate for this purpose. These findings are generally consistent with the approach used in the recent PHERGain Trial [62]. In this trial, patients with early-stage HER2-positive BC were divided into two groups undergoing NAC: (1) receiving non-targeted chemotherapy plus HER2-dual blockade with trastuzumab and pertuzumab, and (2) receiving HER2 dual blockade plus endocrine therapy. All patients underwent baseline [^18^F]FDG PET/CT, and patients in group 2 were further categorized as responders or non-responders based on a ΔSUVmax of -40% in all target lesions after 2 cycles. By applying a ΔSUVmax cut-off of 40% it was possible to identify subjects amenable of chemotherapy de-escalation, in whom an excellent 3-year disease free survival was reached [63]. Similarly to this clinical trial, additional studies should be conducted in patients with TNBC, by using [^18^F]FDG PET/CT as a driver for a personalized NAC treatment.

**Fig. 3:**
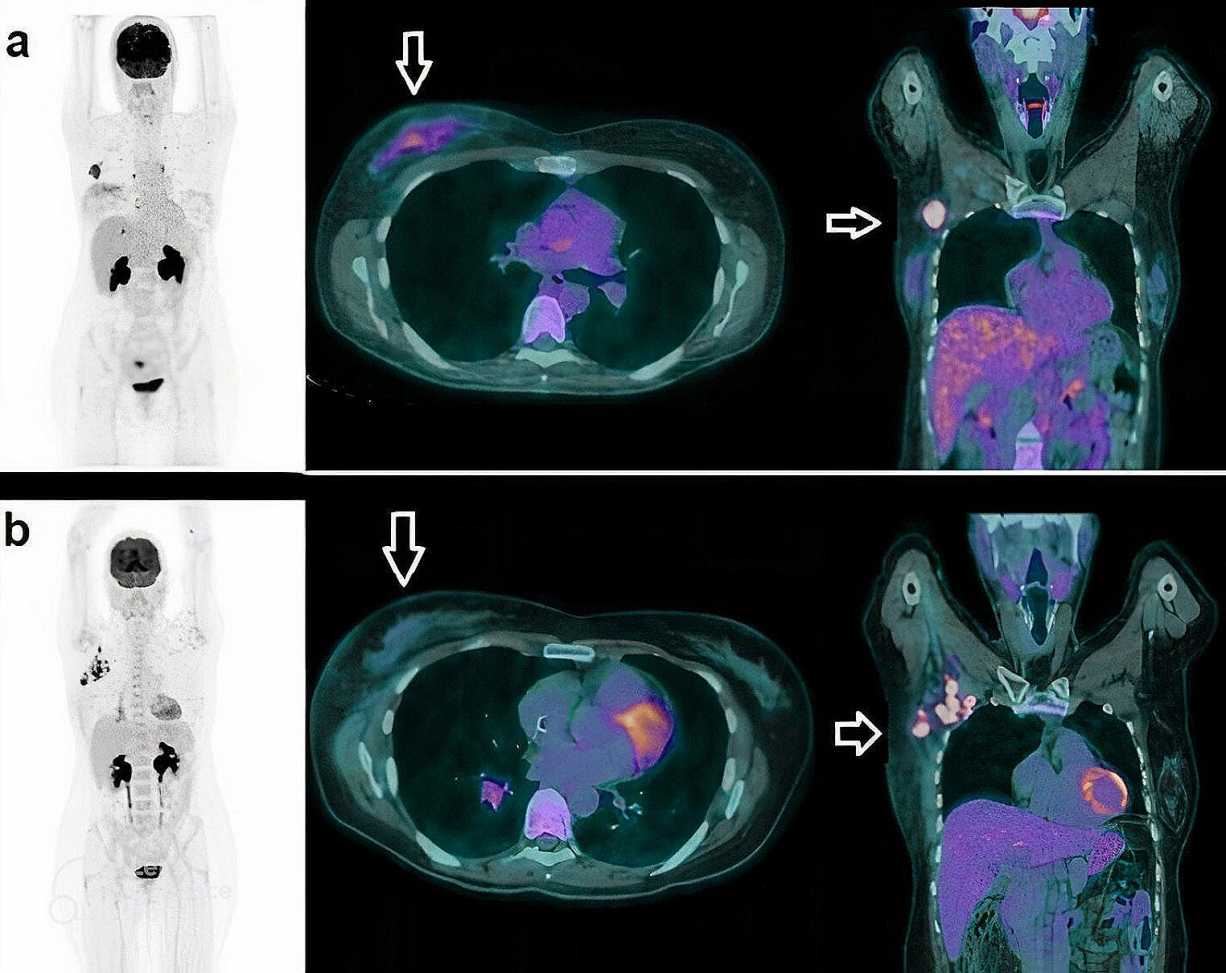
A 42-year-old woman with newly diagnosed triple-negative breast cancer of the right breast. (**a**) upper row: from left to right, whole body PET, axial and coronal slices before NAC showing highly increased tracer uptake within diffuse alteration in right breast (SUVmax 5, arrow) coupled with an enlarged axillary node (SUVmax 9, arrow); (**b**) lower row: from left to right, whole body PET, axial and coronal images after 2 cycles of NAC depicting a reduction in tracer uptake in the right breast (SUVmax 2.7, arrow), in spite of an increased number of pathological axillary nodes (SUVmax 12), as for dissociated response. Patient was classified as non-responder and a therapy switch was carried out

In addition, it should be noted that in the aforementioned studies, response was measured in both primary tumors and axillary lymph nodes. Although uncommon, dissociated responses (where the primary tumor responds while axillary metastases do not, or vice versa) have been reported (Fig. 3), especially in cases involving targeted therapy [64]. The use of [^18^F]FDG PET/CT to identify this specific pattern of response following NAC warrants further investigation.

TNBC recurrence and metastatic disease treatment evaluations were not specifically discussed in our manuscript. Research in these areas typically involves mixed patient cohorts and did not meet our selection criteria. However, these are crucial applications of PET/CT in TNBC, and should be further considered.

For recurrence, histology can influence imaging choices. Invasive lobular carcinoma (often ER + and HER2-) shows low [^18^F]FDG avidity and may benefit from ER-targeted imaging [65]. Invasive ductal carcinomas typically show higher [^18^F]FDG uptake and can be evaluated through metabolic imaging. In such a case, [^18^F]FDG PET/CT is superior to contrast enhanced (ce) CT for detecting recurrence, significantly changing patient management [66]. [^18^F]FDG PET/CT is especially useful for detecting tumor recurrence in multiple sites in asymptomatic patients with elevated tumor markers [66]. A study by Vogsen et al. on 225 women reported a 91% overall diagnostic accuracy for [^18^F]FDG PET/CT in recurrence detection, but did not stratify results by cancer subtype, including TNBC [67].

Metastatic TNBC has limited treatment options and a median OS of 10 months. Accurate therapy response monitoring is crucial. [^18^F]FDG PET/CT is particularly useful for bone-dominant metastases, correlating changes in metabolic activity with outcomes [68]. A study involving 65 patients found that metabolic response assessed by PERCIST was a better predictor of disease-specific survival than CT-based response [69]. Another study highlighted [^18^F]FDG PET/CT’s value in detecting progression earlier than ce CT in 87 women with metastatic BC, including 9 with TNBC [70]. The results obtained in patients with metastatic and recurrent BC support the role of [^18^F]FDG PET/CT in this clinical setting. However, further studies are needed to specifically define the role of metabolic imaging in recurrent or metastatic TNBC.

Two studies have investigated the role of [^18^F]FDG-based radiomics in the context of TNBC [48, 49]. Both studies included relatively small cohorts of patients (*n* = 86 and *n* = 111, respectively) and focused on different topics. Both studies are preliminary and, although encouraging, require further investigation with larger cohorts and well-designed clinical trials [18]. One particularly promising application is the potential of radiomics to identify patients at high risk of relapse, who may therefore require close follow-up or personalized therapeutic regimens. To advance radiomics studies, it would be beneficial to collect data from various clinical centers, as TNBC is a relatively infrequent BC subtype. However, multicenter cooperation necessitates the harmonization of data acquired with different tomographs, also raising issues related to the collection, sharing, and processing of sensitive patient information, which can only be partially anonymized [71].

Finally, PSMA has been tested as an alternative radiopharmaceutical agent in only 2 papers [54, 58], reporting its potential utility both as a diagnostic agent, but also opening to an additional therapeutic strategy in this aggressive disease. From the current data, few comments can be addressed: (1) PSMA can detect more lesions than [^18^F]FDG and (2) it can be correlated with the expression of adverse biomarkers. However, due to the limited available information, few comments can be made, although the rationale can be promising for further research.

Considering the high incidence of BC and its socio-epidemiological impact, the role of PET imaging has been explored in several systematic reviews and meta-analyses [72, 73]. Specifically, the diagnostic performance of PET with various tracers ([^18^F]FDG and [^18^F]NaF) has been compared with that of bone scintigraphy. Metabolic imaging with [^18^F]FDG has proven to be the preferable modality due to its sensitivity, specificity, and ability to identify extra-skeletal locations [72]. Another meta-analysis, which reviewed 29 studies involving 4276 patients, compared the impact of [^18^F]FDG PET alone, PET/CT, and PET/MRI in the staging of BC. The authors found relative proportions of intermodality and intention-to-treat changes induced by PET imaging to be 74% and 70%, respectively [73]. However, all these studies included mixed cohorts of patients with various BC subtypes. The heterogeneity of the included populations led to less conclusive results, and consequently, PET imaging has not been fully integrated into international guidelines or current clinical practice.

The intrinsic value of the present paper, compared to previously published ones, lies in highlighting the potential of [^18^F]FDG PET specifically in the context of TNBC, the rarest and deadliest BC subtype. Additionally, it outlines the promising applications of PSMA-targeted imaging and radiomic approaches. However, the present systematic review has some limitations. First, only studies focused on TNBC were included, excluding those with mixed populations. This choice aligns with the review’s aim to assess the current data available for patients with TNBC alone. Second, the quality of the studies varied. Many papers were retrospective (*n* = 20), and only eight were prospective. Additionally, the methodologies and findings were inconsistent in some cases, without a clear message to share with the community. This highlights the importance of conducting well-designed prospective clinical trials, including in the diagnostic field. Finally, in certain disease settings, such as metastatic TNBC, the results are limited, preventing definitive conclusions.

## Conclusions

In TNBC, baseline [^18^F]FDG PET/CT is a valuable predictive and prognostic biomarker. [^18^F]FDG uptake correlates with tumor aggressiveness and sensitivity to targeted therapies, such as AR inhibitors and immunotherapy. It significantly affects staging, especially in stage II/III patients, and can alter prognosis through the “Will-Rogers phenomenon.” Despite limited data on its impact on final outcomes, its role in assessing response to NAC is particularly promising, but it should be standardized. Additionally, PSMA shows potential as a diagnostic and therapeutic tool, highlighting the need for further research to enhance TNBC management.

## References

[1] Global Cancer Observatory. https://gco.iarc.fr/en. Accessed 30 May 2024.

[2] Foulkes WD, Smith IE, Reis-Filho JS. Triple-negative breast Cancer. N Engl J Med. 2010;363:1938–48. 21067385 10.1056/NEJMra1001389

[3] Li X, Yang J, Peng L, Sahin AA, Huo L, Ward KC, et al. Triple-negative breast cancer has worse overall survival and cause-specific survival than non-triple-negative breast cancer. Breast Cancer Res Treat. 2017;161:279–87. 27888421 10.1007/s10549-016-4059-6

[4] Lehmann BD, Pietenpol JA. Identification and use of biomarkers in treatment strategies for triple-negative breast cancer subtypes. J Pathol. 2014;232:142–50. 24114677 10.1002/path.4280 PMC4090031

[5] Lu B, Natarajan E, Balaji Raghavendran HR, Markandan UD. Molecular classification, treatment, and genetic biomarkers in Triple-negative breast Cancer: a review. Technol Cancer Res Treat. 2023;22. 10.1177/15330338221145246 PMC9829998 36601658

[6] Robson ME, Tung N, Conte P, Im SA, Senkus E, Xu B, et al. OlympiAD final overall survival and tolerability results: Olaparib versus chemotherapy treatment of physician’s choice in patients with a germline BRCA mutation and HER2-negative metastatic breast cancer. Ann Oncol. 2019;30:558–66. 30689707 10.1093/annonc/mdz012 PMC6503629

[7] Geng P, Chi Y, Yuan Y, Yang M, Zhao X, Liu Z, et al. Novel chimeric antigen receptor T cell-based immunotherapy: a perspective for triple-negative breast cancer. Front Cell Dev Biol. 2023;11:1158539. 37457288 10.3389/fcell.2023.1158539 PMC10339351

[8] Sarikaya I. PET receptor imaging in breast cancer. Clin Transl Imaging. 2024;12:5–13.

[9] Llombart-Cussac A, Prat A, Pérez-García JM, Mateos J, Pascual T, Escrivà-de-Romani S, et al. Clinicopathological and molecular predictors of [18F]FDG-PET disease detection in HER2-positive early breast cancer: RESPONSE, a substudy of the randomized PHERGain trial. Eur J Nucl Med Mol Imaging. 2024;51:2733–43. 38587643 10.1007/s00259-024-06683-0 PMC11224085

[10] Loibl S, André F, Bachelot T, Barrios CH, Bergh J, Burstein HJ, et al. Early breast cancer: ESMO Clinical Practice Guideline for diagnosis, treatment and follow-up ☆. Ann Oncol. 2024;35:159–82. 38101773 10.1016/j.annonc.2023.11.016

[11] Vaz SC, Woll JPP, Cardoso F, Groheux D, Cook GJR, Ulaner GA, et al. Joint EANM-SNMMI guideline on the role of 2-[18F]FDG PET/CT in no special type breast cancer: (endorsed by the ACR, ESSO, ESTRO, EUSOBI/ESR, and EUSOMA). Eur J Nucl Med Mol Imaging. 2024;51:2706–32. 38740576 10.1007/s00259-024-06696-9 PMC11224102

[12] Groheux D, Vaz SC, Ulaner GA, Cook GJR, Woll JPP, Mann RM, et al. Joint EANM-SNMMI guidelines on the role of 2-[18F]FDG PET/CT in no special type breast cancer: differences and agreements with European and American guidelines. Eur J Nucl Med Mol Imaging. 2024;51:2701–5. 38693453 10.1007/s00259-024-06694-x

[13] Groheux D, Espié M, Giacchetti S, Hindié E. Performance of FDG PET/CT in the clinical management of breast cancer. Radiology. 2013;266:388–405. 23220901 10.1148/radiol.12110853

[14] Baba S, Isoda T, Maruoka Y, Kitamura Y, Sasaki M, Yoshida T, et al. Diagnostic and prognostic value of pretreatment SUV in 18F-FDG/PET in breast Cancer: comparison with apparent diffusion coefficient from Diffusion-Weighted MR Imaging. J Nucl Med. 2014;55:736–42. 24665089 10.2967/jnumed.113.129395

[15] Koo HR, Park JS, Kang KW, Han W, Park IA, Moon WK. Correlation between 18F-FDG uptake on PET/CT and prognostic factors in triple-negative breast cancer. Eur Radiol. 2015;25:3314–21. 25903708 10.1007/s00330-015-3734-z

[16] Urso L, Quartuccio N, Caracciolo M, Evangelista L, Schirone A, Frassoldati A, et al. Impact on the long-term prognosis of FDG PET/CT in luminal-A and luminal-B breast cancer. Nucl Med Commun. 2022;43:212–9. 35022378 10.1097/MNM.0000000000001500 PMC10876173

[17] Lauri C, Chiurchioni L, Russo VM, Zannini L, Signore A. PSMA expression in solid tumors beyond the prostate gland: Ready for Theranostic Applications? J Clin Med 2022. 2022;11(6590):11:6590. 10.3390/jcm11216590 PMC9657217 36362824

[18] Urso L, Manco L, Castello A, Evangelista L, Guidi G, Castellani M, et al. PET-Derived Radiomics and Artificial intelligence in breast Cancer: a systematic review. Int J Mol Sci 2022. 2022;23(13409):23:13409. 10.3390/ijms232113409 PMC9653918 36362190

[19] Tchou J, Sonnad SS, Bergey MR, Basu S, Tomaszewski J, Alavi A, et al. Degree of tumor FDG uptake correlates with proliferation index in triple negative breast cancer. Mol Imaging Biol. 2010;12:657–62. 20012701 10.1007/s11307-009-0294-0

[20] Lee R, Lee HB, Paeng JC, Choi H, Whi W, Han W, et al. Association of androgen receptor expression with glucose metabolic features in triplenegative breast cancer. PLoS ONE. 2022;17(9):e0275279. 36178912 10.1371/journal.pone.0275279 PMC9524647

[21] Lee HJ, Lim HS, Ki SY, Park HM, Lee JE, Jeong WG, et al. 18F-fluorodeoxyglucose uptake on PET/computed tomography in association with androgen receptor expression and other clinicopathologic factors in surgically resected triple-negative breast cancer. Nucl Med Commun. 2021;42:101–6. 33044403 10.1097/MNM.0000000000001300

[22] Choi SH, Chang JS, Koo JS, Park JW, Sohn JH, Keum KC, et al. Differential prognostic impact of strong PD-L1 expression and 18 F-FDG uptake in triple-negative breast cancer. Am J Clin Oncology: Cancer Clin Trials. 2018;41:1049–57. 10.1097/COC.0000000000000426 29419531

[23] Kimura Y, Sasada S, Emi A, Masumoto N, Kadoya T, Arihiro K, et al. 18F-fluorodeoxyglucose Positron Emission Tomography/Computed Tomography predicts Tumor Immune Microenvironment function in early triple-negative breast Cancer. Anticancer Res. 2023;43:127–36. 36585209 10.21873/anticanres.16141

[24] Denkert C, von Minckwitz G, Darb-Esfahani S, Lederer B, Heppner BI, Weber KE, et al. Tumour-infiltrating lymphocytes and prognosis in different subtypes of breast cancer: a pooled analysis of 3771 patients treated with neoadjuvant therapy. Lancet Oncol. 2018;19:40–50. 29233559 10.1016/S1470-2045(17)30904-X

[25] Xie Y, Liu C, Zhao Y, Gong C, Li Y, Hu S, et al. Heterogeneity derived from 18F-FDG PET/CT predicts immunotherapy outcome for metastatic triple-negative breast cancer patients. Cancer Med. 2022;11:1948–55. 35275444 10.1002/cam4.4522 PMC9089221

[26] Yue Y, Cui X, Bose S, Audeh W, Zhang X, Fraass B. Stratifying triple-negative breast cancer prognosis using 18F-FDG-PET/CT imaging. Breast Cancer Res Treat. 2015;153:607–16. 26346756 10.1007/s10549-015-3558-1 PMC4589560

[27] Choi BB, Lee JS, Kim KH. Association between MRI features and standardized uptake value of 18F-FDG PET/CT in Triple-negative breast Cancer. Oncol Res Treat. 2018;41:706–11. 30321870 10.1159/000492341

[28] Ulaner GA, Castillo R, Goldman DA, Wills J, Riedl CC, Pinker-Domenig K, et al. 18F-FDG-PET/CT for systemic staging of newly diagnosed triple-negative breast cancer. Eur J Nucl Med Mol Imaging. 2016;43:1937–44. 27129866 10.1007/s00259-016-3402-9 PMC5480318

[29] Riedl CC, Slobod E, Jochelson M, Morrow M, Goldman DA, Gonen M, et al. Retrospective analysis of 18F-FDG PET/CT for staging asymptomatic breast Cancer patients younger than 40 years. J Nucl Med. 2014;55:1578–83. 25214641 10.2967/jnumed.114.143297 PMC4414239

[30] Groheux D, Hindié E, Delord M, Giacchetti S, Hamy AS, De Bazelaire C, et al. Prognostic impact of 18 FDG-PET-CT findings in clinical stage III and IIB breast Cancer. JNCI: J Natl Cancer Inst. 2012;104:1879–87. 23243198 10.1093/jnci/djs451 PMC3525816

[31] Conforti F, Pala L, Sala I, Oriecuia C, De Pas T, Specchia C, et al. Evaluation of pathological complete response as surrogate endpoint in neoadjuvant randomised clinical trials of early stage breast cancer: systematic review and meta-analysis. BMJ. 2021;375:e066381. 34933868 10.1136/bmj-2021-066381 PMC8689398

[32] Seban RD, Arnaud E, Loirat D, Cabel L, Cottu P, Djerroudi L, et al. [18F]FDG PET/CT for predicting triple-negative breast cancer outcomes after neoadjuvant chemotherapy with or without pembrolizumab. Eur J Nucl Med Mol Imaging. 2023;50:4024–35. 37606858 10.1007/s00259-023-06394-y

[33] Bouron C, Mathie C, Morel O, Seegers V, Guillerminet C, Lacoeuille F, et al. Correlation between baseline 18F-FDG PET/CT features and pathological complete response after neoadjuvant chemotherapy in early triple negative breast cancer. Med Nucleaire. 2021;45:135–41.

[34] Humbert O, Riedinger JM, Charon-Barra C, Berriolo-Riedinger A, Desmoulins I, Lorgis V, et al. Identification of biomarkers including 18FDG-PET/CT for early prediction of response to neoadjuvant chemotherapy in triple-negative breast cancer. Clin Cancer Res. 2015;21:5460–8. 26130460 10.1158/1078-0432.CCR-15-0384

[35] Groheux D, Biard L, Giacchetti S, Teixeira L, Hindié E, Cuvier C, et al. 18F-FDG PET/CT for the early evaluation of response to neoadjuvant treatment in triple-negative breast cancer: influence of the chemotherapy regimen. J Nucl Med. 2016;57:536–43. 26697967 10.2967/jnumed.115.163907

[36] Groheux D, Hindié E, Giacchetti S, Delord M, Hamy AS, De Roquancourt A, et al. Triple-negative breast cancer: early assessment with 18F-FDG PET/CT during neoadjuvant chemotherapy identifies patients who are unlikely to achieve a pathologic complete response and are at a high risk of early relapse. J Nucl Med. 2012;53:249–54. 22241914 10.2967/jnumed.111.094045

[37] Basnet B, Goyal P, Mahawar V, Bothra S, Agrawal C, Thapa B, et al. Role of18F-flurodeoxyglucose positron-emission tomography/computed tomography in the evaluation of early response to neoadjuvant chemotherapy in patients with locally advanced triple-negative breast cancer. Indian J Nuclear Med. 2020;35:105–9. 10.4103/ijnm.IJNM_210_19 PMC7182325 32351263

[38] Kiyoto S, Sugawara Y, Hosokawa K, Nishimura R, Yamashita N, Ohsumi S, et al. Predictive ability of 18F-fluorodeoxyglucose Positron Emission Tomography/computed tomography for Pathological Complete Response and prognosis after Neoadjuvant Chemotherapy in Triple-negative breast Cancer patients. Asia Ocean J Nucl Med Biol. 2016;4:3. 27904868 10.7508/aojnmb.2016.04.002 PMC4937677

[39] Groheux D, Giacchetti S, Delord M, de Roquancourt A, Merlet P, Hamy AS, et al. Prognostic impact of 18F-FDG PET/CT staging and of pathological response to neoadjuvant chemotherapy in triple-negative breast cancer. Eur J Nucl Med Mol Imaging. 2015;42:377–85. 25432784 10.1007/s00259-014-2941-1

[40] Groheux D, Biard L, Lehmann-Che J, Teixeira L, Bouhidel FA, Poirot B, et al. Tumor metabolism assessed by FDG-PET/CT and tumor proliferation assessed by genomic grade index to predict response to neoadjuvant chemotherapy in triple negative breast cancer. Eur J Nucl Med Mol Imaging. 2018;45:1279–88. 29616304 10.1007/s00259-018-3998-z

[41] Humbert O, Riedinger JM, Vrigneaud JM, Kanoun S, Dygai-Cochet I, Berriolo-Riedinger A, et al. 18F-FDG PET-Derived tumor blood flow changes after 1 cycle of neoadjuvant chemotherapy predicts outcome in triple-negative breast cancer. J Nucl Med. 2016;57:1707–12. 27103025 10.2967/jnumed.116.172759

[42] Marinelli B, Espinet-Col C, Ulaner GA, Mcarthur HL, Gonen M, Jochelson M, et al. Prognostic value of FDG PET/CT-based metabolic tumor volumes in metastatic triple negative breast cancer patients. Am J Nucl Med Mol Imaging. 2016;6(2):120–7. 27186439 PMC4858608

[43] Kim Yil, Kim YJ, Paeng JC, Cheon GJ, Lee DS, Chung JK, et al. Prediction of breast cancer recurrence using lymph node metabolic and volumetric parameters from 18F-FDG PET/CT in operable triple-negative breast cancer. Eur J Nucl Med Mol Imaging. 2017;44:1787–95. 28616695 10.1007/s00259-017-3748-7

[44] Evangelista L, Urso L, Caracciolo M, Stracuzzi F, Panareo S, Cistaro A, et al. FDG PET/CT Volume-Based Quantitative Data and survival analysis in breast Cancer patients: a systematic review of the literature. Curr Med Imaging. 2022;19:807–16. 10.2174/1573405618666220329094423 35352652

[45] Xie Y, Gu B, Hu X, Zhang Y, Zhang J, Wang Z, et al. Heterogeneity of targeted lung lesion predicts platinum-based first-line therapy outcomes and overall survival for metastatic triple-negative breast cancer patients with lung metastasis: a PET biopsy method. Cancer Manag Res. 2019;11:6019–27. 31308743 10.2147/CMAR.S204364 PMC6612961

[46] Gong C, Ma G, Hu X, Zhang Y, Wang Z, Zhang J, et al. Pretreatment 18F-FDG uptake heterogeneity predicts treatment outcome of first-line chemotherapy in patients with metastatic triple-negative breast Cancer. Oncologist. 2018;23:1144–52. 30082489 10.1634/theoncologist.2018-0001 PMC6263118

[47] Soussan M, Orlhac F, Boubaya M, Zelek L, Ziol M, Eder V, et al. Relationship between Tumor Heterogeneity measured on FDG-PET/CT and pathological prognostic factors in invasive breast Cancer. PLoS ONE. 2014;9:e94017. 24722644 10.1371/journal.pone.0094017 PMC3983104

[48] Romeo V, Kapetas P, Clauser P, Baltzer PAT, Rasul S, Gibbs P et al. A simultaneous multiparametric 18F-FDG PET/MRI Radiomics Model for the diagnosis of Triple negative breast Cancer. Cancers (Basel). 2022;14. 10.3390/cancers14163944 PMC9406327 36010936

[49] Bouron C, Mathie C, Seegers V, Morel O, Jézéquel P, Lasla H, et al. Prognostic value of metabolic, volumetric and textural parameters of baseline [18 F]FDG PET/CT in early triple-negative breast Cancer. Cancers (Basel). 2022;14(3):637. 35158904 10.3390/cancers14030637 PMC8833829

[50] Kinoshita Y, Kuratsukuri K, Landas S, Imaida K, Rovito PM, Wang CY, et al. Expression of prostate-specific membrane Antigen in Normal and Malignant Human tissues. World J Surg. 2006;30:628–36. 16555021 10.1007/s00268-005-0544-5

[51] De Galiza Barbosa F, Queiroz MA, Nunes RF, Costa LB, Zaniboni EC, Marin JFG, et al. Nonprostatic diseases on PSMA PET imaging: a spectrum of benign and malignant findings. Cancer Imaging. 2020;20:1–23. 10.1186/s40644-020-00300-7 PMC7071711 32169115

[52] Erhamamcı S, Aslan N. Comparative findings between 68Ga-PSMA and 18F-FDG PET/CT for Hepatocellular Carcinoma. Mol Imaging Radionucl Ther. 2020;29:135. 33094578 10.4274/mirt.galenos.2020.50455 PMC7583742

[53] Urso L, Castello A, Rocca GC, Lancia F, Panareo S, Cittanti C, et al. Role of PSMA-ligands imaging in renal cell carcinoma management: current status and future perspectives. J Cancer Res Clin Oncol. 2022;148:1299–311. 35217902 10.1007/s00432-022-03958-7 PMC9114025

[54] Medina-Ornelas S, García-Perez F, Estrada-Lobato E, Ochoa-Carrillo F. 68Ga-PSMA PET/CT in the evaluation of locally advanced and metastatic breast cancer, a single center experience. Am J Nucl Med Mol Imaging. 2020;10:135. 32704404 PMC7364382

[55] Unger C, Bronsert P, Michalski K, Bicker A, Juhasz-Böss I. Expression of Prostate Specific Membrane Antigen (PSMA) in breast Cancer. Geburtshilfe Frauenheilkd. 2022;82:50–8. 35027860 10.1055/a-1638-9429 PMC8747897

[56] Andryszak N, Świniuch D, Wójcik E, Ramlau R, Ruchała M, Czepczyński R. Head-to-Head Comparison of [18F]PSMA-1007 and [18F]FDG PET/CT in Patients with Triple-Negative Breast Cancer. Cancers 2024, Vol 16, Page 667. 2024;16:667. 10.3390/cancers16030667 PMC10854516 38339419

[57] Katayama A, Handa T, Komatsu K, Togo M, Horiguchi J, Nishiyama M, et al. Expression patterns of claudins in patients with triple-negative breast cancer are associated with nodal metastasis and worse outcome. Pathol Int. 2017;67:404–13. 28699235 10.1111/pin.12560

[58] Arslan E, Ergül N, Beyhan E, Erol Fenercioglu Ö, Sahin R, Cin M, et al. The roles of 68Ga-PSMA PET/CT and 18F-FDG PET/CT imaging in patients with triple-negative breast cancer and the association of tissue PSMA and claudin 1, 4, and 7 levels with PET findings. Nucl Med Commun. 2023;44:284–90. 36756767 10.1097/MNM.0000000000001663

[59] Hildebrandt MG, Naghavi-Behzad M, Vogsen M. A role of FDG-PET/CT for response evaluation in metastatic breast cancer? Semin Nucl Med. 2022;52:520–30. 35525631 10.1053/j.semnuclmed.2022.03.004

[60] O’Reilly D, Sendi M, Al, Kelly CM. Overview of recent advances in metastatic triple negative breast cancer. World J Clin Oncol. 2021;12:164. 33767972 10.5306/wjco.v12.i3.164 PMC7968109

[61] Al-Mahmood S, Sapiezynski J, Garbuzenko OB, Minko T. Metastatic and triple-negative breast cancer: challenges and treatment options. Drug Delivery Translational Res 2018. 2018;8:5. 10.1007/s13346-018-0551-3 PMC6133085 29978332

[62] Pérez-García JM, Gebhart G, Ruiz Borrego M, Stradella A, Bermejo B, Schmid P, et al. Chemotherapy de-escalation using an 18F-FDG-PET-based pathological response-adapted strategy in patients with HER2-positive early breast cancer (PHERGain): a multicentre, randomised, open-label, non-comparative, phase 2 trial. Lancet Oncol. 2021;22:858–71. 34019819 10.1016/S1470-2045(21)00122-4

[63] Pérez-García JM, Cortés J, Ruiz-Borrego M, Colleoni M, Stradella A, Bermejo B, et al. 3-year invasive disease-free survival with chemotherapy de-escalation using an 18F-FDG-PET-based, pathological complete response-adapted strategy in HER2-positive early breast cancer (PHERGain): a randomised, open-label, phase 2 trial. Lancet. 2024;403:1649–59. 38582092 10.1016/S0140-6736(24)00054-0

[64] Humbert O, Chardin D. Dissociated response in metastatic Cancer: an atypical pattern brought into the spotlight with immunotherapy. Front Oncol. 2020;10:566297. 33072599 10.3389/fonc.2020.566297 PMC7531255

[65] Mankoff D, Balogova S, Dunnwald L, Dehdashti F, DeVries E, Evangelista L, et al. Summary: SNMMI Procedure Standard/EANM Practice Guideline for Estrogen Receptor Imaging of patients with breast Cancer using 16α-[18F]Fluoro-17β-Estradiol PET. J Nucl Med. 2024;65:221–3. 38071554 10.2967/jnumed.123.266938

[66] Radan L, Ben-Haim S, Bar-Shalom R, Guralnik L, Israel O. The role of FDG-PET/CT in suspected recurrence of breast cancer. Cancer. 2006;107:2545–51. 17063499 10.1002/cncr.22292

[67] Vogsen M, Jensen JD, Gerke O, Jylling AMB, Asmussen JT, Christensen IY, et al. Benefits and harms of implementing [18F]FDG-PET/CT for diagnosing recurrent breast cancer: a prospective clinical study. EJNMMI Res. 2021;11:1–11. 34553294 10.1186/s13550-021-00833-3 PMC8458550

[68] Tateishi U, Gamez C, Dawood S, Yeung HWD, Cristofanilli M, Macapinlac HA. Bone metastases in patients with metastatic breast cancer: morphologic and metabolic monitoring of response to systemic therapy with integrated PET/CT. Radiology. 2008;247:189–96. 18372468 10.1148/radiol.2471070567

[69] Riedl CC, Pinker K, Ulaner GA, Ong LT, Baltzer P, Jochelson MS, et al. Comparison of FDG-PET/CT and contrast-enhanced CT for monitoring therapy response in patients with metastatic breast cancer. Eur J Nucl Med Mol Imaging. 2017;44:1428–37. 28462446 10.1007/s00259-017-3703-7 PMC5526620

[70] Vogsen M, Harbo F, Jakobsen NM, Nissen HJ, Dahlsgaard-Wallenius SE, Gerke O, et al. Response monitoring in metastatic breast cancer – a prospective study comparing 18F-FDG PET/CT with conventional CT. J Nucl Med. 2022;64:355–61. 36207136 10.2967/jnumed.121.263358 PMC10071809

[71] Stamhuis E, Evangelista L, van der Voort S, Mele AM, Spiller E, Demir E, et al. From bottleneck to enabler: a new approach to regulating data-driven medical research. Clin Transl Imaging. 2023;11:311–3.

[72] Zamanian M, Treglia G, Abedi I. Diagnostic accuracy of PET with different Radiotracers versus Bone Scintigraphy for detecting bone metastases of breast Cancer: a systematic review and a Meta-analysis. J Imaging. 2023;9:274. 38132692 10.3390/jimaging9120274 PMC10744045

[73] Han S, Choi JY. Impact of 18F-FDG PET, PET/CT, and PET/MRI on staging and management as an initial staging modality in breast Cancer: a systematic review and Meta-analysis. Clin Nucl Med. 2021;46:271. 33651022 10.1097/RLU.0000000000003502 PMC7938917

